# Differential expression of microRNA, miR-150 and enhancer of zeste homolog 2 (EZH2) in peripheral blood cells as early prognostic markers of severe forms of dengue

**DOI:** 10.1186/s12929-020-0620-z

**Published:** 2020-01-18

**Authors:** Harsha Hapugaswatta, Pubudu Amarasena, Ranjan Premaratna, Kapila N. Seneviratne, Nimanthi Jayathilaka

**Affiliations:** 1 Department of Chemistry, Faculty of Science, University of Kelaniya, Kelaniya, Sri Lanka; 2 North Colombo Teaching Hospital, Ragama, Sri Lanka; 3 Department of Medicine, Faculty of Medicine, University of Kelaniya, Kelaniya, Sri Lanka

**Keywords:** Dengue, Severe dengue, microRNA, Acute dengue biomarkers

## Abstract

**Background:**

Dengue presents a wide clinical spectrum. Most patients recover following a self-limiting non-severe clinical course. A small proportion of patients progress to severe disease, mostly characterized by plasma leakage with or without hemorrhage. Early symptoms of severe dengue (SD) are similar to those of non-severe dengue fever (DF). Severe symptoms manifest after 3–5 days of fever, which can be life threatening due to lack of proper medications and inability to distinguish severe cases during the early stages. Early prediction of SD in patients with no warning signs who may later develop severe infection is very important for proper disease management to alleviate related complications and mortality. microRNA are small non-coding RNA molecules that regulate post-transcriptional gene expression. Due to the remarkable stability and the role of microRNA in gene expression, altered expression of microRNA was evaluated to explore clinically relevant prognostic markers of severe dengue.

**Methods:**

The relative expression of microRNA hsa-let-7e (let-7e), hsa-miR-30b-5p (miR-30b), hsa-miR-30e-3p (miR-30e), hsa-miR-33a (miR-33a), and hsa-miR-150-5p (miR-150) and several putative target genes in peripheral blood cells (PBC) collected from 20 DF and 20 SD positive patients within 4 days from fever onset was evaluated by quantitative reverse transcription PCR (qRT-PCR).

**Results:**

miR-150 showed significant (*P* < 0.01) up regulation in PBC of SD patients compared to DF patients during the acute phase of infection. Expression of enhancer of zeste homolog 2 (EZH2) was significantly (*P* < 0.01) down regulated indicating that genes involved in epigenetic regulation are also differentially expressed in SD patients during the early stage of infection.

**Conclusions:**

Differential expression of microRNA miR-150 and the putative target gene EZH2 may serve as reliable biomarkers of disease severity during early stages of dengue infection.

## Background

More than half of the world population is at risk of contracting dengue, a mosquito-borne viral infection with approximately 50 to 100 million cases reported each year in many tropical countries including Sri Lanka [1]. In 2018, 51,659 suspected dengue cases have been reported to the Epidemiology Unit of Sri Lanka from all over the island making it the most important tropical disease posing a significant public health threat. DF is characterized by fever with two of the following criteria: nausea, vomiting, rash, aches and pains, positive tourniquet test, leukopenia with or without warning signs. The warning signs include, abdominal pain or tenderness, persistent vomiting, clinical fluid accumulation, mucosal bleeding, lethargy, restlessness, liver enlargement > 2 cm, increase in hematocrit level (HCT) concurrent with rapid decrease in platelet count. Severe manifestations of dengue also show similar symptoms during the early stages of infection. After 3–5 days from fever onset, SD patients manifest severe plasma leakage leading to: shock, fluid accumulation with respiratory distress and severe bleeding with severe organ involvement (liver: aspartate aminotransferase (AST) or alanine aminotransferase (ALT) ≤1000, central nervous system (CNS): impaired consciousness, heart and other organs). The degree of increase above the baseline HCT often reflects the severity of plasma leakage [2]. Humans possessing pre-infection antibody, passively acquired or derived from heterotypic infection are susceptible to SD [3]. There are four dengue virus (DENV) serotypes each of which is capable of infection [4]. DENV1 and DENV2 have been reported as prevalent serotypes with a similar number of reported infections [5]. All four virus serotypes are present in Sri Lanka. DENV2 and DENV3 are the common serotypes reported in many parts of Sri Lanka, with DENV3 reported to be responsible for many of the infections that progress to SD [6]. Inability to distinguish SD from DF during the early stages of infection makes this disease life threatening.

There are no proper medications or vaccines available for prevention and treatment of dengue [7]. The first ever dengue vaccination program was launched in 2016 in Philipines with complications rising from increased risk of developing SD in particular circumstances [1]. Therefore, dengue prevention is largely limited to vector control. Several research groups have reported potential small molecules for drug intervention of dengue [8–10]. In addition, there is ongoing research on antiviral activity of phytochemicals as well as currently available antiviral drugs against dengue viruses. However, these phytochemicals and drugs have not been approved for the management of dengue infections [11, 12]. Diagnostic tests based on PCR or serological testing for dengue do not distinguish SD from DF and disease severity is determined after the patient is presented with severe symptoms [13]. However, if diagnosed early, effective disease management only involves hospital care and hydration to mitigate complications from SD [14]. Since there are no early clinical tests approved for prognosis of SD, most patients suspected of having contracted dengue are hospitalized for disease management resulting in high healthcare costs, though only a small fraction of DF patients develop SD. Therefore, identification of markers for the early detection of SD could effectively cut down dengue related mortality as well as cost of healthcare during dengue outbreaks.

Several studies comparing the transcriptomes of DF and SD patients have reported increased levels of cytokines, tumor necrosis factor alpha (TNF-α), interleukin 2 (IL-2), interleukin 6 (IL-6), interleukin 10 (IL-10), interleukin 12 (IL-12) and interferon gamma (IFN-γ) involved in host immune responses in SD patients [15–19]. Genetic varients in major histocompatibility complex class I polypeptide-related sequence B (MICB) and 1-phosphatidylinositol-4,5-bisphosphate phosphodiesterase epsilon-1(PLCE1) has been found to be associated with dengue [20]. However, these studies have not determined whether these markers serve as differential markers during the early stage of infection when severe manifestations of dengue cannot be distinguished based on the clinical symptoms.

MicroRNA molecules are small non-coding RNA molecules with the length around 20–22 nucleotides. Due to their role in regulating the expression of putative target genes, microRNAs are important for function of human cells. Therefore, microRNAs show differential expression in different diseases. Since microRNAs are also remarkably stable in blood, they are under investigation as potential biomarkers. Recent studies have reported differential expression of microRNA in dengue patients and infected cultured cells [21, 22]. let-7e, miR-30b and miR-33a have been found to differentially express in DENV infected cultured cells [23]. Similarly, miR-30e also expresses differentially in dengue infected cultured cells [24]. miR-150 expression is upregulated in DENV infected cultured cells within few hours of the infection [25]. Expression of miR-150 is also found to be significantly upregulated in SD patients compared to DF patients [26]. However, the expression levels of miR-150 during the early stages of dengue infection, when prediction of disease outcome is not possible, has not been compared in patient samples to determine the potential to serve as an early indicator of progression to SD. Therefore, we evaluated the relative expression of microRNAs let-7e, miR-30b, miR-30e, miR-33a and miR-150 against the geometric mean of hsa-miR-16-5p (miR-16) and hsa-miR-103a-3p (miR-103a) as reference genes and their putative target genes in PBC from blood samples collected from dengue positive patients within 4 days of fever onset by qRT-PCR.

let-7e and miR-150 have been connected to regulation of EZH2 expression [27]. In addition, miR-30b has been shown to regulate DNA methyl transferase 3 alpha (DNMT3A) expression that can be used as a potential biomarker and a therapeutic target for cancer [28]. DNMT3A has been found to regulate herpes simplex virus-1 propagation [29]. miR-33a regulates the expression of receptor interacting protein 140 (RIP140) and adenosine triphosphate-binding cassette transporter subfamily A member 1 (ABCA1) involved in lipid metabolism and transportation, thus, play a role in viral invasion. miR-33a directly down regulates the expression of ABCA1 in cholesterol metabolism pathway and also contributes to the regulation of cholesterol and fatty acid homeostasis by targeting RIP-140 [30]. There is also evidence that DENV infection leads to an autophagy-dependent processing of lipid droplets and triglycerides to release free fatty acids which increases the cellular *β*-oxidation that generates ATP. These processes are required for efficient DENV replication [31]. ABCA1 expression inhibits Hepatitis C virus cell entry, acting on virus-host cell fusion [32]. Therefore, the relative expression of these putative target genes of above microRNA, EZH2, ABCA1, DNMT3A, and RIP140 were evaluated against glyceraldehyde 3-phosphate dehydrogenase (GAPDH) as the reference gene for their potential to serve as markers of early prognosis of SD.

## Methods

### Sample collection and processing

Adult male and female patients (above age 18) who presented with clinical symptoms of dengue viral infection within 4 days from fever onset were tested onsite for dengue using nonstructural protein 1 (NS1) rapid test (SD Bio) at the North Colombo Teaching Hospital, Sri Lanka. The World Health Organization (WHO) classification for dengue was used to identify patients with the relevant clinical symptoms: fever with two of the following criteria: nausea, vomiting, rash, aches and pains, positive tourniquet test, leukopenia with or without warning signs; the warning signs include, abdominal pain or tenderness, persistent vomiting, clinical fluid accumulation, mucosal bleeding, lethargy, restlessness, liver enlargement > 2 cm, increase in HCT concurrent with rapid decrease in platelet count. Patients who tested positive in the NS1 test were recruited for this retrospective case-control study with informed consent. None of the patients were pregnant. After recruitment, patients who developed SD were determined according to the WHO guidelines based on clinically detectable pleural effusions and ascites as evidence of plasma leakage using a portable bedside ultrasonogram [2]. None of the patients presented signs of SD at the time of recruitment within 4 days from fever onset. Twenty patients presented with signs of SD after recruitment during the course of infection. Samples collected at the time of recruitment within 4 days from fever onset from the patients who later presented signs of SD were selected as the severe cases. Twenty samples collected at the time of admission within 4 days from fever onset from patients who did not present with signs of SD during the course of infection were randomly selected as controls with non-severe dengue. None of the patients recruited for the study succumbed to death from dengue during the course of infection. A 2.5 ml ethylenediaminetetraacetic acid (EDTA) blood sample was collected from patients at the time of admission within 4 days from fever onset and transported and processed at 4 °C within 2 h from sample collection. Isolated PBC were stored at − 80 °C until sample analysis.

### qRT-PCR

MicroRNA primers were designed based on the miRbase database and gene specific human primers against EZH2, DNMT3A, ABCA1 and the reference gene, GAPDH were mined from previously published literature (Additional file 1: Tables S1 and S2). Primers for microRNA target genes, were designed using NCBI primer picker to span exon-exon junctions. Total RNA was isolated from PBC using miRNeasy Serum/Plasma kit (Qiagen) according to the manufacturer’s instructions. cDNA was synthesized using the miScript II RT Kit with Hiflex buffer for polyadenylation of microRNA according to product manual (Qiagen). Expression of both microRNA and mRNA target genes was quantified using miScript SYBR Green PCR kit at an annealing temperature of 58 °C for microRNA and 60 °C for mRNA according to product manual (Qiagen). All targets under investigation amplified earlier than 35 cycles. Each reaction was carried out in triplicates in 20 μL reaction volume using StepOne Real-Time PCR System (Applied Bio Systems). The efficiency of amplification was above 80% based on the standard curve analysis. No-template reactions and melting curve analysis was used to confirm specificity of target amplification. Expression levels of the individual miRNAs relative to the geometric mean of the expression level of reference miRNAs and the expression levels of individual target genes relative to the expression level of the reference gene in DF and SD patients were calculated as 2^−ΔCq^. Relative expression of miRNAs and target genes are shown as log_2_ values. Continuous variables were expressed as median and interquartile range (IQ_25–75_). The fold change of microRNA and mRNA expression was calculated using the eq. 2^-ΔΔCq^ and presented as log_2_ values.

### Statistical analysis

q-q plots and Shapiro-Wilk test were used to determine normality at a 95% confidence interval. For the Shapiro-Wilk test *P* > 0.05 was determined as normal distribution. Statistically significant differences among the median ± median absolute deviation (MAD) was determined using Mann-Whitney U test for non-parametric independent samples. A *P* < 0.05 based on the Mann-Whitney U test was considered a statistically significant difference. Statistical significance for differentially expressed targets were determined using independent t-test with Bonferroni adjusted α value of 0.01. *P* < 0.01 was considered a statistically significant difference based on standard error of mean (SEM) of ∆Cq. Logistic regression analysis for odds ratio, receiver operator characteristics, area under curve, specificity and sensitivity was determined using IBM SPSS Statistics, 2013 version at a 95% confidence interval.

## Results

### Differential expression analysis of selected microRNA in dengue patients within 4 days from fever onset

Expression of let-7e, miR-30b, miR-30e, miR-33a and miR-150 in PBC collected at admission, within 4 days from fever onset from patients confirmed to have presented with DF and patients who later developed SD based on clinical symptoms was analyzed. The data were analyzed against the geometric mean of miR-103a and miR-16 reported to have stable expression in PBC during infections. Patients were classified as DF based on the clinical symptoms. Patients who later developed SD were classified based on evidence of plasma leakage (pleural effusions and ascites) detected using a portable ultrasonogram. Twenty DF and twenty SD samples were analyzed. Among them, most of the subjects were male (77%), with a median age of 30 (18–60) while the female subjects had a median age of 24 (19–60) years. At enrollment, there were no statistically significant differences in median laboratory clinical parameters including platelet count, HCT, AST and ALT levels in patients who later developed SD compared with DF (Table 1). All patients classified as SD based on evidence of plasma leakage also presented with platelet count below 100,000/mm^3^ after day 4 from fever onset. None of the patients recruited for the study presented with HCT above the baseline (> 52% for male and > 48% for female) during the course of infection. While both DF patients and, patients who later developed SD presented with an increase in HCT after day 5, only patients who later developed SD presented with a significantly higher (*P* < 0.05) HCT after day 5 (Additional file 1: Table S3). Therefore, DF patients could not be distinguished from the SD patients during the acute febrile phase within 4 days from fever onset based on the clinical characteristics.

**Table 1:** Clinical characteristics of dengue patients recruited for gene expression analysis at admission (median ± MAD)

	DF patients (*n* = 20)	SD patients (*n* = 20)	*P* value
Gender (Male%/Female%)	70/30	85/15	
Age	30 (18–60)	27 (19–60)	
Platelet (×1000 /mm^3^)	119.0 ± 39.0	125.0 ± 24.0	0.66
Hematocrit (%)	40.0 ± 3.1	39.7 ± 2.2	0.57
Hemoglobin (g/dL)	14.0 ± 1.0	13.0 ± 1.2	0.15
White blood cells (×1000 cells/mm^3^)	3.2 ± 0.6	4.3 ± 0.9	0.07
Neutrophils (%)	66.3 ± 9.7	75.0 ± 10.0	0.14
Lymphocytes (%)	27.0 ± 10.0	17.0 ± 7.0	0.10
Eosinophils (%)	0.6 ± 0.4	1.0 ± 1.0	0.84
AST (U/L)	32.0 ± 20.0	58.5 ± 18.0	0.06
ALT (U/L)	29.0 ± 14.0	43.1 ± 11.4	0.11

**Fig. 1:**
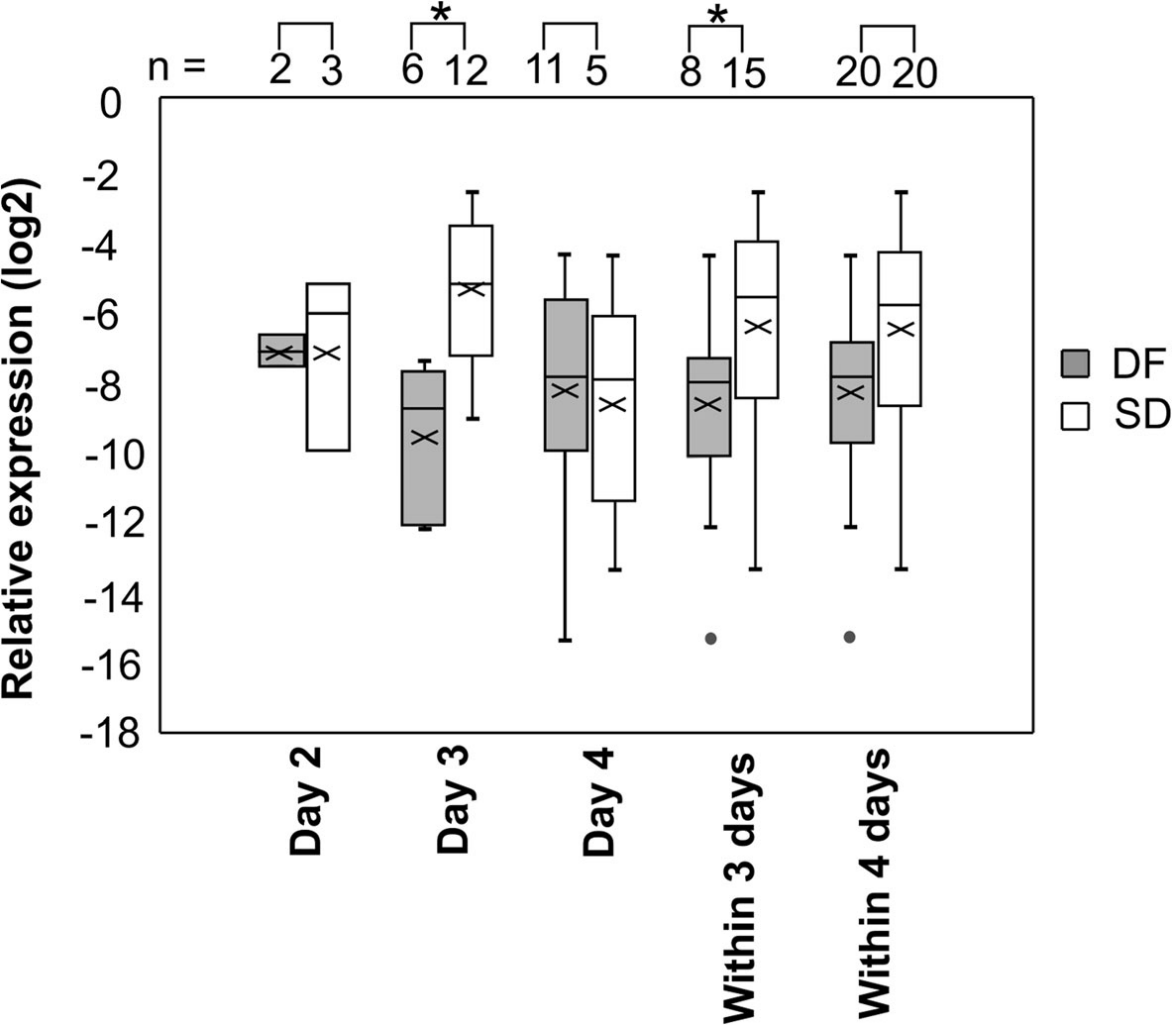
Relative miR-150 expression in PBC samples in DF and SD patients. Relative expression at admission in patients recruited on, day 2 (n_DF_ = 2, n_SD_ = 3), day 3 (n_DF_ = 6, n_SD_ = 12), day 4 (n_DF_ = 11, n_SD_ = 5), all patients recruited within 3 days (n_DF_ = 8, n_SD_ = 15) and within 4 days (n_DF_ = 20, n_SD_ = 20) from fever onset. Relative expression presented as log values to the base 2 based on 2^−ΔCq^ values against geometric mean of miR-16 and miR-103a. * *P* < 0.01 based on ΔCq ± SEM using independent t – test with Bonferroni adjustment

The data for microRNA expression in all patient samples at admission, collected within 4 days from fever onset (either on day 2, day 3 or day 4 from fever onset), are normally distributed at 95% confidence interval. miR-150 expression showed significant (*P* < 0.01) upregulation at admission in samples collected from patients recruited on day 3 (n_DF_ = 6, n_SD_ = 12) and all samples collected at admission from patients recruited within 3 days (on day 2 and day 3) (n_DF_ = 8, n_SD_ = 15) from fever onset before the patients present with symptoms of severe illness. Samples collected at admission from patients recruited on day 2 (n_DF_ = 2, n_SD_ = 3) and day 4 (n_DF_ = 11, n_SD_ = 5) from fever onset failed to show differential expression (Fig. 1, Additional file 1: Table S4). This may be due to limited number of patients recruited within 2 days from fever onset, available for expression analysis at admission. The rest of the microRNA under investigation did not show significant differential expression in SD patients over DF patients within 4 days from fever onset (Additional file 2: Figure S1).

Logistic regression analysis for miR-150 expression based on ΔCq values was found to be predictive of SD at admission in all patient samples collected within 3 days from fever onset with odds ratio of 0.52 (95%, CI;0.29–0.93, *P* = 0.03). The area under the receiver operating characteristic curve (AUC) for miR-150 expression at admission is 0.85 (sensitivity 0.80, specificity 0.88) for all patient samples collected within 3 days from fever onset at ΔCq of 7.54 (*P* < 0.05) and 0.92 (sensitivity 1.00, specificity 0.50) for just the patient samples collected on day 3 from fever onset at ΔCq of 9.25 (P < 0.05) indicating that miR-150 may serve as an early marker for development of severe manifestations of dengue within 3 days from fever onset (Additional file 2: Figure S2).

### Differential expression analysis of putative target mRNA of the selected microRNA in dengue patients within 4 days from fever onset

MicroRNAs suppress or inhibit the expression of putative target genes. As such, the upregulation of microRNA expression may lead to the downregulation of the putative target gene expression. Therefore, relative expression of putative target genes of the microRNA under investigation, EZH2, ABCA1, DNMT3A and RIP140 were evaluated against the expression of GAPDH as a reference gene at admission within 4 days from fever onset, in samples collected from DF (*n* = 20) patients and those who later developed SD (*n* = 20).

**Fig. 2:**
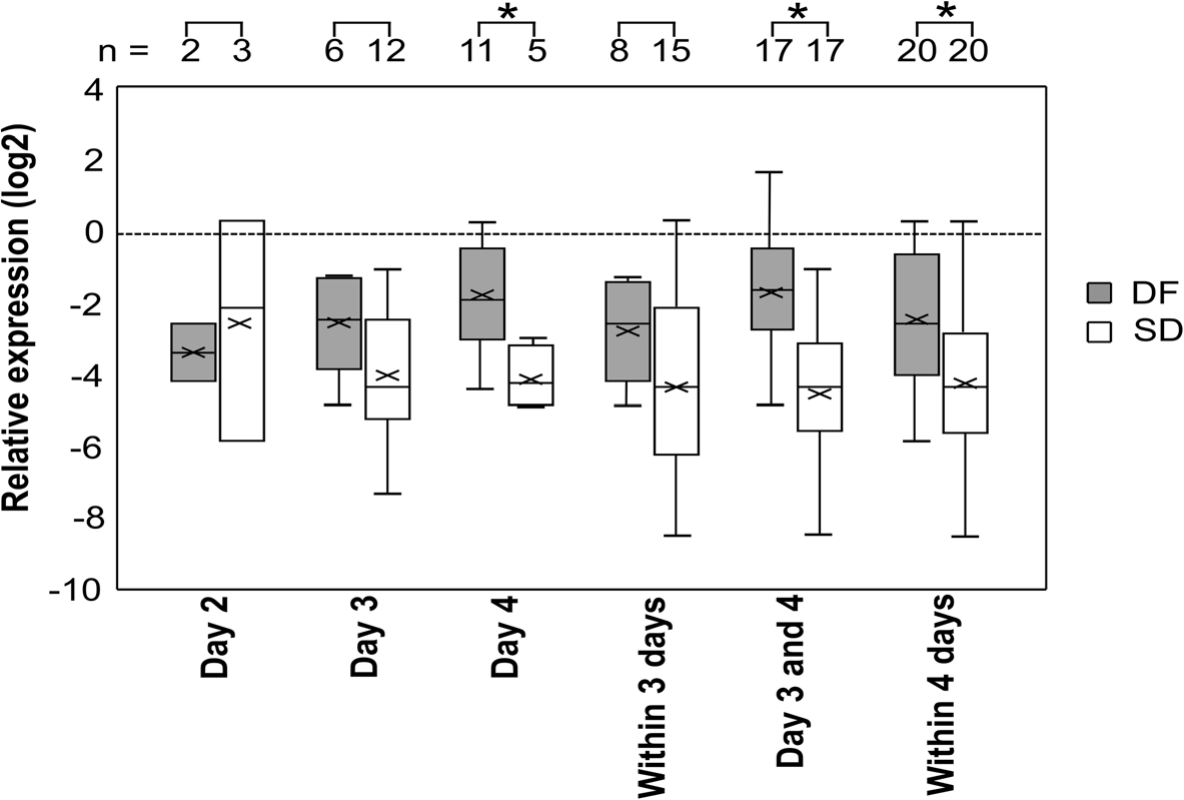
Relative EZH2 expression in PBC from DF and SD patients. Relative expression at admission in patients recruited on, day 2 (n_DF_ = 2, n_SD_ = 3), day 3 (n_DF_ = 6, n_SD_ = 12), day 4 (n_DF_ = 11, n_SD_ = 5), all patients recruited on day 3 & day 4 (n_DF_ = 17, n_SD_ = 17), within 3 days (n_DF_ = 8, n_SD_ = 15) and within 4 days (n_DF_ = 20, n_SD_ = 20) from fever onset presented as log values to the base 2 (log2) based on 2^−ΔCq^ values against GAPDH. * *P* < 0.01 based on ΔCq ± SEM using independent t – test with Bonferroni adjustment

All the data for the target gene expression were normally distributed. EZH2 expression showed significant down regulation (*P* < 0.01) in samples collected from patients who later developed SD compared to that in DF patients at admission within 4 days from fever onset. Out of those patients, evaluation of differential expression at admission in DF and SD samples collected from patients recruited on day 3 (n_DF_ = 6, n_SD_ = 12), day 4 (n_DF_ = 11, n_SD_ = 5) and all patients recruited within 3 days (n_DF_ = 8, n_SD_ = 15) from fever onset also showed a downregulation in EZH2 expression. However, the downregulation in expression was only significant (*P* < 0.01) at admission in samples collected from patients recruited on day 4 (n_DF_ = 11, n_SD_ = 5) from fever onset and all patients recruited on day 3 and day 4 (n_DF_ = 17, n_SD_ = 17) from fever onset (Fig. 2, Additional file 1: Table S5). These findings are consistent with upregulation of miR-150 in PBC of SD patients compared to DF patients at the early stages.

Logistic regression analysis for EZH2 expression at admission in samples collected from patients recruited on day 4 from fever onset is found to be predictive for SD with odds ratio of 4.23 at 95% CI; 0.93–19.34, AUC of 0.91 with sensitivity of 0.60 and specificity of 0.82 at ΔCq of 3.44. Odds ratio for expression at admission in samples collected from all patients recruited on day 3 and day 4 from fever is 2.32 at 95% CI; 1.28–4.18 (*P* = 0.001), AUC of 0.86 with sensitivity of 0.82 and specificity of 0.81 at ΔCq of 2.89 (*P* = 0.00). Odds ratio for expression at admission in samples collected from all patients recruited within 4 days from fever is 1.60 at 95% CI; 1.12–2.31 (*P* = 0.01), AUC of 0.76 with sensitivity of 0.85 and specificity of 0.45 at ΔCq of 2.04 (*P* = 0.01) (Additional file 2: Figure S4). ABCA1, DNMT3A and RIP140 failed to show differential expression within 4 days from fever onset among the DF and SD samples. ABCA1 showed downregulation of expression only among patient samples collected on day 4 from fever onset (Additional file 2: Figure S3).

## Discussion

miR-150 is significantly upregulated at admission (*p* < 0.01) in SD patients recruited on day 3 and all patients recruited within 3 days from fever onset. Samples collected on day 4 did not show significant differential expression in miR-150 at admission among the DF and SD patients. Evaluating differential microRNA expression in a larger cohort of patient samples is needed to evaluate the fluctuations in the level of microRNA from fever onset during the early stages of infection. Augmented miR-150 expression has been reported in patients presented with SD after 4 days of fever [26]. Therefore, expression analysis during defervescence (day 3 to 8) may shed further insight on progression into the critical phase. The expression changes during convalescence when the patients recover from the febrile illness may also shed light on the role of miR-150 during disease progression. Due to logistic limitations and limited patient consent, our study was limited to analysis of the level of expression at admission within 4 days from fever onset when differential diagnosis is not possible. In addition, we were not able to analyze the differences in expression among the DF patients with warning signs and without warning signs due to the limited number of DF samples included in the study. However, miR-150 expression within 3 days from fever onset can be used as a prognostic marker for severity of dengue infection. The expression level of EZH2, a putative target gene of miR-150 is also significantly downregulated in SD patients at the time of admission (*p* < 0.01) within 4 days from fever onset.

miR-150 has been shown to play a crucial role in hematopoiesis. Overexpression of miR-150 has been shown to result in inhibition of erythroid proliferation [33]. However, upregulation of miR-150 expression within 3 days from fever onset in dengue patients who later developed severe manifestations of the infection did not show a corresponding drop in the HCT. In fact, a rise in HCT above the baseline is considered a warning sign for developing SD. Both DF and SD patients recruited for this study presented with a rise in the HCT after day 5 compared to the HCT at admission with SD showing a significant increase (*P* < 0.05). The down regulation of miR-150 expression on 4 days from fever onset in patients who later develop SD may contribute to the rise in the HCT.

Loss of EZH2 has also been shown to inhibit erythropoiesis [34]. However, inhibition of EZH2 protein expression has been shown to induce erythroid differentiation. EZH2 knockdown had shown no significant effect on the expression of erythroid-related genes suggesting an indirect role for EZH2 in regulation of erythroid differentiation [35]. Therefore, significant downregulation of EZH2 expression within 4 days from fever onset in the PBC of patients who later developed SD may also contribute to the significant increase in HCT (*P* < 0.05) in those patients after day 5. Suppression of EZH2 has been correlated with the induction of multiple inflammatory, stress, and antipathogen pathways [36]. EZH2/1 inhibitors have been shown to induce antiviral responses that suppressed infection with DNA (human cytomegalovirus, adenovirus) and RNA (Zika virus) viruses. Inhibition of EZH2 expression repressed the herpes simplex virus infection [35]. Further, inhibition of EZH2 has also been associated with viral repression via interferon/immune signaling-related pathways by mediating proinflammatory responses such as increased IL-6, IL-6 receptor, IFN-α1, IFN-α2 and IL-8 levels, all of which have also been implicated in SD [36, 37].

Hypermethylation of miR-150 promoter was detected in association with suppressed miR-150 expression in the livers of Beta-hydroxy-beta-methylbutyrate-treated piglets [38]. EZH2 is an epigenetic modifier that can catalyze the methylation of histone H3 protein. Epigenetic modifications are hereditary DNA or histone modifications that do not change the DNA sequence but affect transcriptional regulation. Differential expression of epigenetic modifiers such as EZH2 in patients marked by plasma leakage and/or thrombocytopenia suggests that there is a role for epigenetic regulation in severe manifestations of dengue. Therefore, epigenetic modifications may play a role in severe manifestation of dengue during secondary infections. Therefore, significant down regulation of EZH2 within 4 days from fever onset may contribute to the significant upregulation of miR-150 expression within 3 days from fever onset. Further studies in cultured cells may be needed to evaluate the role of miR-150 on EZH2 expression during severe DENV infections.

Regulation of miR-150 and EZH2 expression has also been linked to inducible Nitric Oxide Synthase (iNOS or NOS2) activity where iNOS is indirectly regulated by miR-150. A study showed, that infection of mycobacterium bovis bacillus calmette-guérin regulates the epigenetic changes at class II transactivator (CIITA) promoter via induction of expression of iNOS and miR-150 through the recruitment of the transcription factor. According to our studies, differential expression analysis of iNOS in DF and SD patient samples collected on day 2, 3, 4, within 3 days and within 4 days from fever onset showed downregulation in SD patients as early as day 2 from fever onset and remained downregulated [39].

let-7e, which has been reported to directly regulate EZH2 expression is only upregulated in SD patients on day 2 and day 4 from fever onset while the expression is downregulated on day 3. Differential expression of EZH2 as a result of upregulation of let-7e expression was implicated in several viral infections including DENV2 infected peripheral blood mononuclear cells (PBMCs) [40]. Therefore, further studies in DENV infected cultured cells may be needed to evaluate the role of let-7e on the differential expression of EZH2 during SD.

The polycomb group protein, EZH2 and DNMT3A are both involved in epigenetic regulation via DNA methylation. Binding of DNMTs to several EZH2-repressed genes depends on the presence of EZH2. EZH2 serves as a recruitment platform for DNA methyltransferases [41]. Since both EZH2 and DNMT3A participate in epigenetic modification mediated transcriptional regulation, differential expression analysis in virus infected cultured cells may also elucidate the role of DNMT3A in SD. Expression of ABCA1 and RIP140 implicated in lipid metabolism [32], a mechanism which has been shown to play a role in viral infections on the other hand did not significantly change in patients with SD.

Further, circulating miR-150 levels have been reported to correlate with pro-inflammatory cytokine-induced fever. A significant increase in the circulating miR-150 levels has been reported to associate with fatal outcome in A/H1N1 patients compared to patients that survived the febrile infection [42]. Upregulation of miR-150 in SD patients at the time of admission, within 4 days from fever onset suggests a role similar to other febrile illnesses.

## Conclusions

The expression level of miR-150 is significantly upregulated within 3 days from fever onset in the PBC of patients who later developed severe manifestations. Consequently, expression of the epigentic modifier EZH2 is significantly downregulated in the patients. Therefore, our studies have shown that differential expression of microRNA miR-150 and the putative target gene EZH2 may serve as reliable biomarkers of disease severity during early stages of dengue infection when differential diagnosis or prognosis is not possible based on the clinical evidence.

## Supplementary information

**Additional file 1: Table S1.** Primers for microRNA genes for qRT-PCR analysis. **Table S2.** Primers for microRNA target genes for qRT-PCR analysis. **Table S3.** AST, ALT and HCT of patients from admission to discharge. **Table S4.** Relative expression of microRNA at admission. **Table S5.** Relative expression of putative target genes of microRNA at admission.

**Additional file 2: Figure S1.** Fold change of relative miRNA expression in PBC samples between DF and SD patients, **Figure S2.** ROC curves for miR-150 on (a) day 3, (b) within 3 days from fever onset, **Figure S3.** Fold change of relative microRNA target gene expression in PBC between DF and SD patients, **Figure S4.** ROC curves for EZH2 on (a) day 4, and (b) day 3 & day 4 and (c) within 4 days from fever onset.

## Data Availability

All data generated or analyzed during this study are included in this published article [and its supplementary information files].

## Abbreviations

ABCA1: Adenosine triphosphate-binding cassette transporter subfamily A member 1
ALT: Alanine aminotransferase
AST: Aspartate aminotransferase
CIITA: Class II transactivator
CNS: Central nervous system
DENV: Dengue virus
DF: Non-severe dengue fever
DNMT3A: DNA methyl transferase 3 alpha
EDTA: Ethylenediaminetetraacetic acid
EZH2: Enhancer of zeste homolog 2
GAPDH: Glyceraldehyde 3-phosphate dehydrogenase
HCT: Hematocrit level
IFN-γ: Interferon gamma
IL-10: Interleukin 10
IL-12: Interleukin 12
IL-2: Interleukin 2
IL-6: Interleukin 6
iNOS: inducible Nitric Oxide Synthase
let-7e: hsa-let-7e
log_2_: log values to the base 2
MAD: Median absolute deviation
MICB: Major histocompatibility complex class I polypeptide-related sequence B
miR-103a: hsa-miR-103a-3p
miR-150: hsa-miR-150-5p
miR-16: hsa-miR-16-5p
miR-30b: hsa-miR-30b-5p
miR-30e: hsa-miR-30e-3p
miR-33a: hsa-miR-33a
NS1: Nonstructural protein 1
PBC: Peripheral blood cells
PBMCs: Peripheral blood mononuclear cells
PLCE1: 1-Phosphatidylinositol-4,5-bisphosphate phosphodiesterase epsilon-1
qRT-PCR: quantitative reverse transcription PCR
RIP140: Receptor interacting protein 140
SD: Severe dengue
SEM: Standard error of mean
TNF-α: Tumor necrosis factor alpha
WHO: World Health Organization
