# Supplementary material for: Differential expression of microRNA, miR-150 and enhancer of zeste homolog 2 (EZH2) in peripheral blood cells as early prognostic markers of severe forms of dengue
Source: J Biomed Sci. 2020 Jan 18;27:25. doi: 10.1186/s12929-020-0620-z (PMC6969970; doi:10.1186/s12929-020-0620-z)
Supplement: Supplementary file 1 — Additional file 1: Table S1. Primers for microRNA genes for qRT-PCR analysis. Table S2. Primers for microRNA target genes for qRT-PCR analysis. Table S3. AST, ALT and HCT of patients from admission to discharge. Table S4. Relative expression of microRNA at admission. Table S5. Relative expression of putative target genes of microRNA at admission. [file 12929_2020_620_MOESM1_ESM.docx]

**Table S1. Primers for microRNA genes for qRT-PCR analysis**

| Target gene | Primer sequence (5’ 3’) | miRBase accession code |
| --- | --- | --- |
| miR-16 | TAGCAGCACGTAAATATTGGCG | MIMAT0000069 |
| miR-103a | AGCAGCATTGTACAGGGCTATGA | MIMAT0000101 |
| let-7e | GGGTGAGGTAGGAGGTTGTAT | MI0000066 |
| miR-30b | GGGTGTAAACATCCTACACTCA | MIMAT0000420 |
| miR-30e | CTTTCAGCGGATGTTTACAGC | MI0000091 |
| miR-33a | GGGGTCATTGTAGTTGC | MIMAT0000693 |
| miR-150 | TCTCCCAACCCTTGTACCAGTG | MIMAT0000451 |

**Table S2. Primers for microRNA target genes for qRT-PCR analysis**

| Target genes | Forward primer  (5’🡪3’) | Reverse primer  (5’🡪3’) | NCBI accession code | Publications |
| --- | --- | --- | --- | --- |
| GAPDH | TGCACCACCAACTGCTTAGC | GGCATGGACTGTGGTCATGAG | NM_002046.6 | McCurdy, D. et al. (2008) ‘Validation of the comparative quantification method of real-time PCR analysis and a cautionary tale of housekeeping gene selection’, Gene Ther Mol Biol, 12, pp.15-24. |
| EZH2 | CCCTGACCTCTGTCTTACTTGTGGA | ACGTCAGATGGTGCCAGCAATA | XM_017011817.2 | Fujii, S. et al. (2008) ‘Enhancer of zeste homologue 2 (EZH2) down-regulates RUNX3 by increasing histone H3 methylation’, J Biol Chem, 283(25), pp. 17324–17332. doi: 10.1074/jbc.M800224200. |
| DNMT3A | TATTGATGAGCGCACAAGAGAGC | GGGTGTTCCAGGGTAACATTGAG | NM_175629.2 | Qiu X. et al. (2010) ‘Epigenetic activation of E-cadherin is a candidate therapeutic target in human hepatocellular carcinoma’, Exp Ther Med, 1(3), Pp. 519–523. doi: 10.3892/etm_00000082. |
| ABCA1 | AACAGTTTGTGGCCCTTTTG | AGTTCCAGGCTGGGGTACTT | NM_005502.3 | Sporstøl, M. et al. (2007) ‘ABCA1, ABCG1 and SR-BI: hormonal regulation in primary rat hepatocytes and human cell lines’, BMC Mol Biol, 8(5). doi: 10.1186/1471-2199-8-5. |
| RIP140 | TGGGGAAGTGTTTGGATTGT | TGTGCATCTTCTGGCTGTG | NM_003489.3 | Not applicable |

**Table S3. AST, ALT and HCT of patients from admission to discharge**

| **AST level (U/L), [n]** | | | P value |
| --- | --- | --- | --- |
| Days from fever onset | DF | SD |  |
| At admission | 45.0±16.0, [20] | 61.0±24.5, [20] | 0.44 |
| Day 5 | 73.0±44.0, [9] | 57.5±8.0, [5] | 0.65 |
| Day 6 | 68.5±14.5, [6] | 142.0±0.0, [3] | 0.16 |
| Day 7 | 185.5±42.5, [2] | 115.0±14.2, [2] | 0.63 |
| Day 8 | 417.5±226.5, [2] | 160.0±38.0, [6] | 0.09 |
| Day 5 to 8 | 116.0±52.0, [10] | 142.0±38.0, [11] | 0.61 |
| **ALT level, (U/L), [n]** | | |  |
| Days from fever onset | DF | SD |  |
| At admission | 33.0±14.0, [20] | 47.0±15.0, [20] | 0.44 |
| Day 5 | 53.0±21.5, [8] | 41.7±15.7, [5] | 0.53 |
| Day 6 | 67.0±17.0, [7] | 71.0±9.0, [3] | 0.75 |
| Day 7 | 116.0±57.0, [3] | 80.2±56.1, [3] | 0.38 |
| Day 8 | 212.6±0.0, [1] | 105.5±7.0, [6] | 0.13 |
| Day 5 to 8 | 72.0±28.0, [11] | 100.5±24.0, [12] | 0.71 |
| **HCT (%), [n]** | | | |
| Days from fever onset | DF | SD |  |
| At admission | 40.0±2.2, [14] | 39.8±2.8, [17]^b^ | 0.30 |
| Day 5 | 36.0±5.3, [3] | 45.9±4.3, [7]^a^ | 0.08 |
| Day 6 | 36.4±4.8, [2] | 39.5±4.2, [3] | 0.27 |
| Day 7 | 41.5±1.8, [4] | 43.6±1.4, [3] | 0.49 |
| Day 8 | 47.0±0.0, [1] | 44.6±3.3, [3] | 0.54 |
| Day 5 to 8 | 41.5±8.6, [7] | 43.6±3.5, [9]^b^ | 0.57 |

AST and ALT levels (Median±MAD) for [n] number of patients and HCT % (Median±MAD) (P<0.05 considered as a statistically significant difference using Mann-Whitney U test). Significant differences between at admission and on day 5 and at admission and day 5 to 8 marked by a, b down the column (P<0.05 considered as a statistically significant difference using paired t – test).

**Table S4. Relative expression of microRNA at admission**

| miRNA | Days from fever onset | 2^-^ **^ΔΔ^**^Cq^  (log_2_) | Mean **Δ**Cq ±SEM | | P values |  |
| --- | --- | --- | --- | --- | --- | --- |
|  |  |  | DF | SD |  |  |
| let-7e | Day 2 | 2.15 | 5.54±1.36 | 5.06±0.70 | 0.74 |  |
|  | Day 3 | -1.84 | 4.81±0.58 | 5.65±0.72 | 0.53 |  |
|  | Day 4 | 1.30 | 5.58±0.38 | 4.43±0.71 | 0.14 |  |
|  | Within 3 days | -0.79 | 5.05±0.46 | 5.57±0.60 | 0.27 |  |
|  | Within 4 days | 0.24 | 5.34±0.27 | 5.27±0.50 | 0.91 |  |
| miR-30b | Day 2 | 1.42 | 10.89±0.91 | 9.46±1.52 | 0.54 |  |
|  | Day 3 | -0.40 | 10.18±1.08 | 10.57±0.60 | 0.74 |  |
|  | Day 4 | 0.28 | 9.85±0.64 | 9.60±0.71 | 0.81 |  |
|  | Within 3 days | 0.01 | 10.36±0.82 | 10.35±0.55 | 0.99 |  |
|  | Within 4 days | 0.04 | 10.19±0.47 | 10.16±0.45 | 0.96 |  |
| miR-30e | Day 2 | 0.69 | 14.36±0.83 | 13.67±0.34 | 0.49 |  |
|  | Day 3 | 1.30 | 15.57±1.01 | 14.26±0.59 | 0.25 |  |
|  | Day 4 | -0.22 | 14.55±0.85 | 14.79±0.67 | 0.86 |  |
|  | Within 3 days | 1.12 | 15.26±0.78 | 14.14±0.48 | 0.21 |  |
|  | Within 4 days | 0.36 | 14.66±0.54 | 14.30±0.39 | 0.60 |  |
| miR-33a | Day 2 | 1.82 | 15.25±0.32 | 13.42±2.65 | 0.53 |  |
|  | Day 3 | 1.69 | 17.77±1.10 | 16.08±1.09 | 0.35 |  |
|  | Day 4 | -2.06 | 16.63±0.92 | 18.69±1.36 | 0.23 |  |
|  | Within 3 days | 1.59 | 17.14±0.91 | 15.55±1.02 | 0.32 |  |
|  | Within 4 days | 0.25 | 16.58±0.62 | 16.33±0.87 | 0.82 |  |
| miR-150 | Day 2 | 0.06 | 7.35±0.45 | 7.30±1.45 | 0.98 |  |
|  | Day 3 | 4.04 | 9.68±0.86 | 5.64±0.65 | 0.00* |  |
|  | Day 4 | -0.38 | 8.42±0.91 | 8.83±1.44 | 0.81 |  |
|  | Within 3 days | 3.13 | 9.10±0.74 | 5.97±0.60 | 0.00* |  |
|  | Within 4 days | 1.76 | 8.55±0.59 | 6.68±0.62 | 0.04 |  |

Relative expression of microRNA presented as fold change based on 2^-ΔΔCq^ values against miR-16 and miR-103a (log_2_) and **Δ**Cq at 95% confidence intervals (CI). * P<0.01 considered as statistical significant differential expression based on ΔCq ± SEM using independent t – test with Bonferroni adjustment**.**

**Table S5.** **Relative expression of putative target genes of microRNA at admission**

Relative expression presented as fold change based on 2^-ΔΔCq^ values against GAPDH (log_2_) and **Δ**Cq at 95% confidence intervals (CI). * P<0.01 considered as statistical significant differential expression based on ΔCq ± SEM using independent t – test with Bonferroni adjustment**.**

| Putative target genes | Days from fever onset | 2^-^ **^ΔΔ^**^Cq^  (log_2_) | Mean **Δ**Cq±SEM | | P values |  |
| --- | --- | --- | --- | --- | --- | --- |
|  |  |  | DF | SD |  |  |
| EZH2 | Day 2 | 0.83 | 3.40±0.81 | 2.57±1.51 | 0.76 |  |
|  | Day 3 | -2.18 | 3.39±0.81 | 4.78±0.67 | 0.07 |  |
|  | Day 4 | -2.74 | 1.31±0.56 | 4.06±0.38 | 0.01* |  |
|  | Within 3 days | -1.51 | 2.81±0.51 | 4.33±0.68 | 0.12 |  |
|  | Day 3 & day 4 | -2.86 | 1.70±0.45 | 4.57±0.51 | 0.00* |  |
|  | Within 4 days | -2.06 | 2.20±0.44 | 4.26±0.51 | 0.00* |  |
| DNMT3A | Day 2 | -0.15 | 4.24±1.09 | 3.85±0.29 | 0.96 |  |
|  | Day 3 | 0.89 | 3.55±0.99 | 4.55±0.62 | 0.90 |  |
|  | Day 4 | -1.29 | 2.86±0.47 | 3.60±0.34 | 0.30 |  |
|  | Within 3 days | 0.30 | 3.75±0.74 | 4.41±0.53 | 0.90 |  |
|  | Day 3 & day 4 | -0.57 | 3.09±0.45 | 4.27±0.50 | 0.51 |  |
|  | Within 4 days | -0.04 | 3.36±0.39 | 4.20±0.43 | 0.62 |  |
| ABCA1 | Day 2 | 0.39 | 1.20±1.61 | 1.35±1.46 | 0.69 |  |
|  | Day 3 | -1.00 | 3.71±1.27 | 3.55±0.63 | 0.39 |  |
|  | Day 4 | -0.58 | 2.65±0.52 | 3.66±0.88 | 0.31 |  |
|  | Within 3 days | 0.56 | 2.87±1.05 | 3.04±0.77 | 0.48 |  |
|  | Day 3 & day 4 | -1.18 | 3.09±0.56 | 3.58±0.66 | 0.09 |  |
|  | Within 4 days | -0.79 | 2.75±0.54 | 3.15±0.62 | 0.16 |  |
| RIP140 | Day 2 | -0.07 | -0.99±2.48 | -0.91±2.57 | 0.99 |  |
|  | Day 3 | -1.51 | 1.16±1.11 | 2.70±0.82 | 0.31 |  |
|  | Day 4 | -0.92 | 1.47±0.67 | 1.79±1.35 | 0.81 |  |
|  | Within 3 days | -0.43 | 0.55±1.02 | 1.98±0.70 | 0.27 |  |
|  | Day 3 & day 4 | -1.07 | 1.36±0.56 | 2.43±0.74 | 0.28 |  |
|  | Within 4 days | -0.92 | 1.36±0.55 | 1.92±0.74 | 0.54 |  |
