# Supplementary material for: Differential expression of microRNA, miR-150 and enhancer of zeste homolog 2 (EZH2) in peripheral blood cells as early prognostic markers of severe forms of dengue
Source: J Biomed Sci. 2020 Jan 18;27:25. doi: 10.1186/s12929-020-0620-z (PMC6969970; doi:10.1186/s12929-020-0620-z)
Supplement: Supplementary file 2 — Additional file 2: Figure S1. Fold change of relative miRNA expression in PBC samples between DF and SD patients, Figure S2. ROC curves for miR-150 on (a) day 3, (b) within 3 days from fever onset, Figure S3. Fold change of relative microRNA target gene expression in PBC between DF and SD patients, Figure S4. ROC curves for EZH2 on (a) day 4, and (b) day 3 & day 4 and (c) within 4 days from fever onset. [file 12929_2020_620_MOESM2_ESM.docx]

**
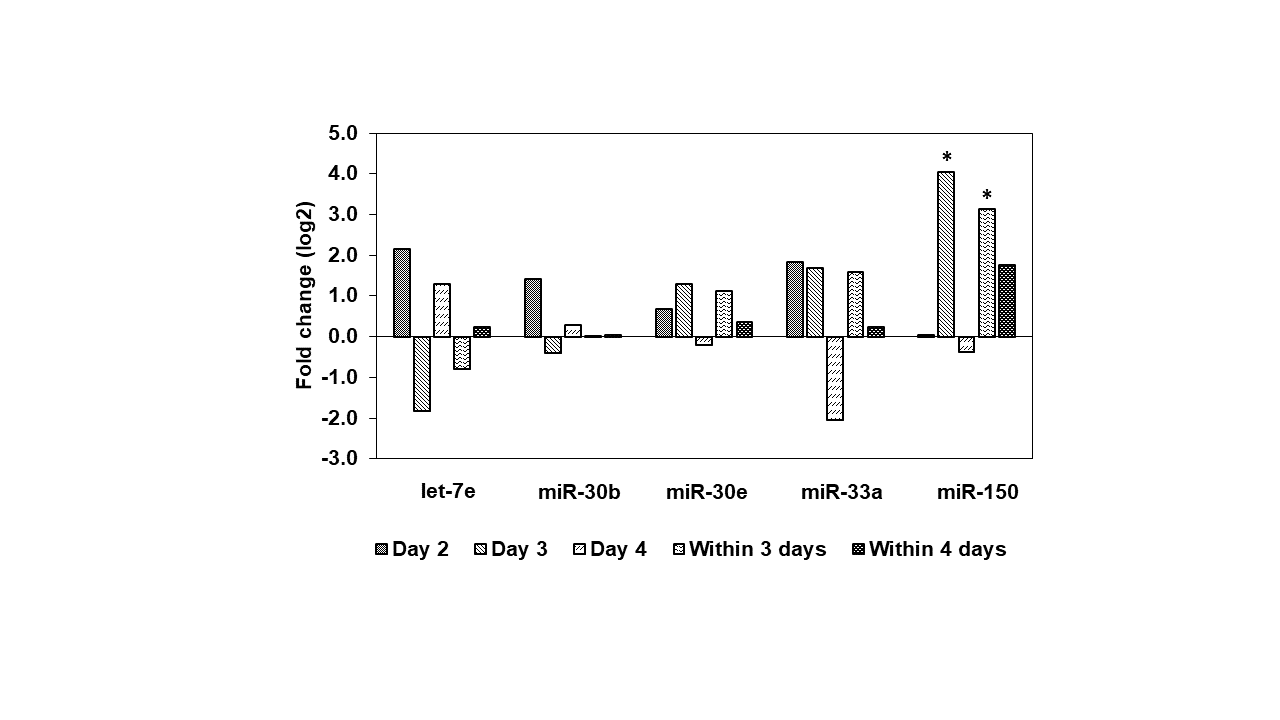
**

**Figure S1.** Fold change of relative miRNA expression in PBC samples between DF and SD patients. Fold change of relative expression at admission in patients recruited on, day 2 (n_DF_=2, n_SD_ =3), day 3 (n_DF_=6, n_SD_=12), day 4 (n_DF_=11, n_SD_=5), within 3 days (n_DF_=8, n_SD_=15) and within 4 days (n_DF_=20, n_SD_=20) from fever onset. Fold change of expression based on 2^-ΔΔCq^ values against geometric mean of miR-16 and miR-103a, where a fold change >1.5 was considered as up regulation and < 0.5 considered as down regulation presented as log_2_ values. * P<0.01 based on ΔCq ± SEM using independent t – test with Bonferroni adjustment.


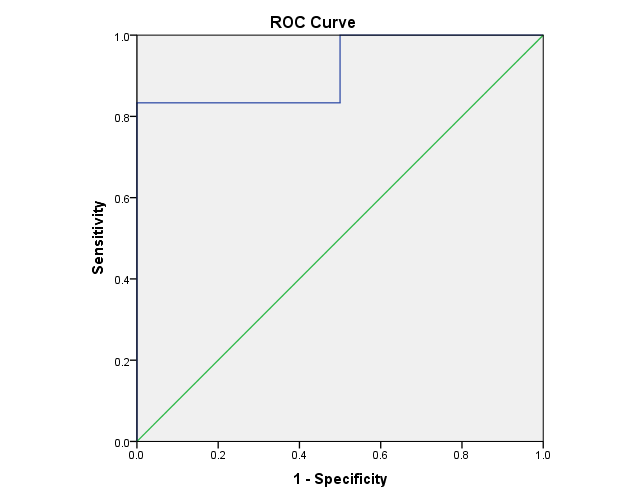


**b**

**a**


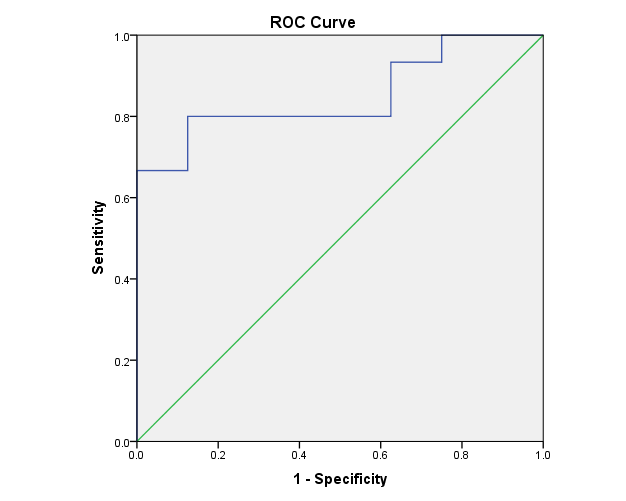


**Figure S2.** ROC curves for miR150 on (a) day 3, (b) within 3 days from fever onset.

**
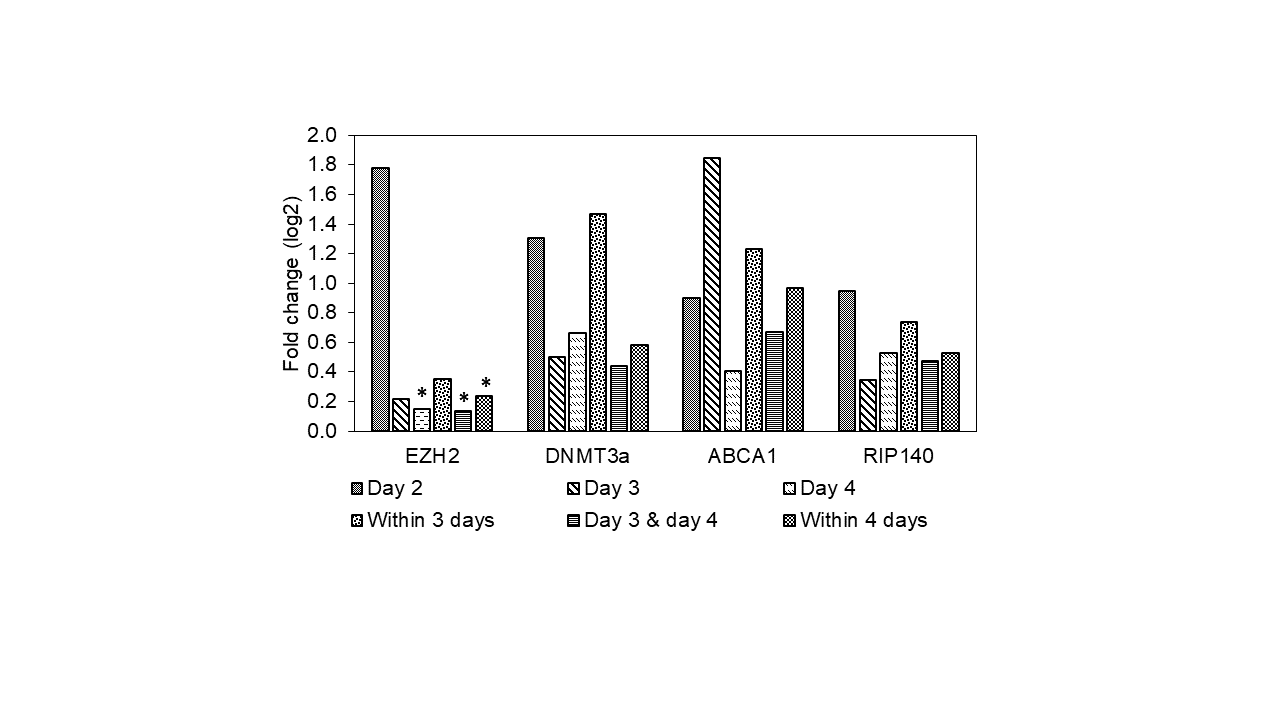
**

**Figure S3.** Fold change of relative microRNA target gene expression in PBC between DF and SD patients. Fold change of relative expression at admission in patients recruited on, day 2 (n_DF_=2, n_SD_ =3), day 3 (n_DF_=6, n_SD_=12), day 4 (n_DF_=11, n_SD_=5), within 3 days (n_DF_=8, n_SD_=15), day 3 & day 4 (n_DF_=17, n_SD_=17) and within 4 days (n_DF_=20, n_SD_=20) from fever onset based on 2^-ΔΔCq^ values against GAPDH, where a fold change >1.5 was considered as upregulation and < 0.5 considered as downregulation presented as log_2_ values. * P<0.01 based on ΔCq ± SEM using independent t – test with Bonferroni adjustment.


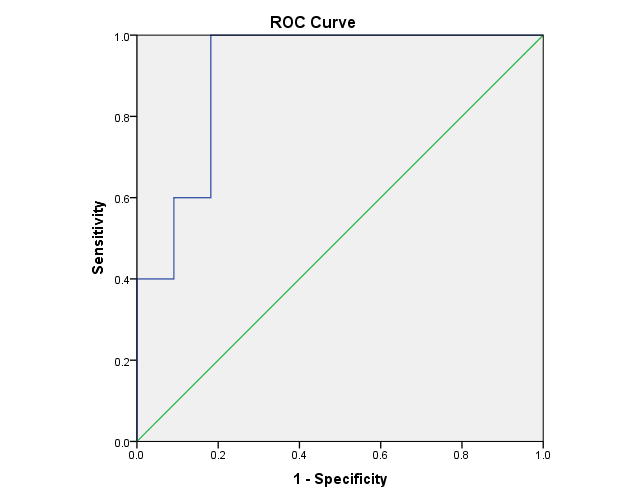


**a**


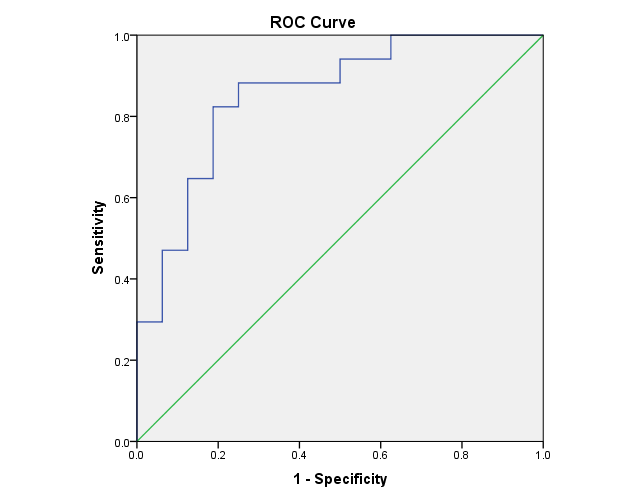


**b**


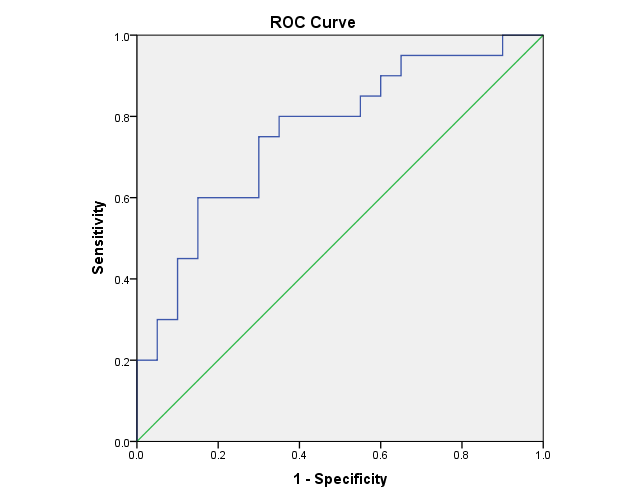


**c**

**Figure S4.** ROC curves for EZH2 on (a) day 4 (b) day 3 & day 4 (c) within 4 days from fever onset.
